# The effects of high-intensity interval training versus moderate-intensity continuous training on fat loss and cardiometabolic health in pediatric obesity: A protocol of systematic review and meta-analysis

**DOI:** 10.1097/MD.0000000000014751

**Published:** 2019-03-08

**Authors:** Jing-Xin Liu, Lin Zhu, Jia-Min Deng

**Affiliations:** a Research Center for Physical Fitness and Health Promotion of Adolescent, Guangzhou Sport University, Guangzhou, China; b School of Kinesiology, Shanghai University of Sport, Shanghai, China.

**Keywords:** cardiometabolic health, childhood obesity, fat loss, high-intensity interval training, moderate-intensity continuous training

## Abstract

**Background:**

The effects of aerobic exercise on fat loss and cardiometabolic health are well-documented, but it is unknown whether a high-intensity interval training (HIIT) elicit a greater health benefit in obese children and adolescents.

**Methods:**

Relevant studies in Pubmed, Web of Science, Embase, the Cochrane Library, EBSCO, and CNKI will be searched for studies with language restriction in English and Chinese, which were published from inception to December 1, 2018. Only randomized controlled trials of HIIT on pediatric obesity will be included, and observational studies, prospective cohort studies, and systematic reviews will be excluded. Two reviewers will independently screen the studies; risk of bias assessment and data extraction, and the results are inconsistent when discussed or resolved by a third reviewer. Data analysis and synthesis will be completed by the Revman 5.3 software and Stata 12.0 software. This study will be conducted by following the guideline of the Preferred Reporting Items for Systematic Review and Meta-analysis Protocols.

**Conclusion:**

This study will be conducted by previously published data, thus ethics approval is not required. This finding will be published in a related peer-reviewed journal and present it at international conferences.

**PROSPERO registration number:**

CRD42018111308,

## 1 Introduction

Childhood obesity, defined by the World Health Organization (WHO) as abnormal or excessive fat accumulation that presents a risk to health, is one of the most serious global public health challenges of the 21st century. The latest epidemiological studies have found that there were 107.7 million children who were obese in the world in 2015, and the growth rate of childhood obesity has been greater than the growth rate of adult obesity in many countries.^[1]^ Strong evidences have shown that excess weight during childhood is a predictor of future obesity, with high incidence of noncommunicable diseases such as cardiovascular disease, type 2 diabetes, musculoskeletal diseases, and some cancers at early younger age.^[2,3]^ In fact, cardiovascular disease risk factor, such as insulin resistance, dyslipidemia, hypertension, and poor cardiorespiratory fitness in obese children, are becoming more common. In particular, poor cardiorespiratory fitness is considered to be an important predictor of cardiovascular disease, which has attracted more attention in recent research.^[4,5]^

Physical activity is a critical component in managing childhood obesity, and it has been recommended by both the American College of Sports Medicine and WHO that children and adolescents should perform at least 60 minutes per day of moderate-to-vigorous physical activity (MVPA), and at least 3 times per week of high-intensity exercise. In pediatric population, the traditional methods to increase physical activity have principally used continuous training, which trend to rely on longer-duration sessions involving moderate-intensity continuous training (MICT).^[6,7]^ Although previous studies have examined that MICT is associated with significant reduction in body fat and cardiovascular risk factor in obese children and adolescents, studies have shown that the majority of children failed to meet the recommended physical activity guidelines, such as only 30% of school-aged children met MVPA recommendations in China.^[8]^ Lacking of time has been considered as main barriers. Therefore, it is necessary to exploring more time-efficient exercise modality for obese children and adolescents.

High-intensity interval training (HIIT), defined as alternate short bursts of high-intensity exercise and light exercise or passive recovery periods, has been considered as an alternative and time-efficient strategy to MICT. The emerging evidence has revealed that HIIT could lead to greater or comparable abdominal and visceral fat mass loss and cardiorespiratory fitness improvement than MICT among overweight and obese adults,^[9,10]^ and could more effectively reduce metabolic risk factors in type 2 diabetes, indicating that HIIT may be a superior modality to keep cardiometabolic health.^[11,12]^ Furthermore, a meta-analysis conducted by Wewege et al.^[13]^ showed that short-term HIIT could elicit modest improvement in body fat and waist circumference in overweight and obese adults, which can reduce 40% times commitment each week compared with MICT. However, HIIT performed in obese children and adolescents is significantly less investigated than adults and chronic disease patients; it still remains uncertain whether it is the best form of exercise for obese children and adolescents to lose fat and maintain cardiometabolic health. Therefore, the aim of this systematic review with meta-analysis was to analyze the published studies that investigated the effects of HIIT versus MICT on fat loss and cardiometabolic health in pediatric obesity.

### 1.2 Review objectives

We expect to try to answer the following questions through meta-analysis: whether HIIT is effective in losing weight and reducing cardiovascular risk factors, and whether it is more effective than MICT. A better understanding of the differences between HIIT and MICT on weight loss and health benefits in childhood obesity will better inform development of exercise intervention strategy to prevention and treatment of childhood obesity.

## 2 Methods

### 2.1 Protocol and registration

The protocol of this meta-analysis followed the guideline of the Preferred Reporting Items for Systematic Review and Meta-Analysis Protocols (PRISMA-P),^[14]^ and this protocol has been registered in Prospective Register of Systematic Reviews (registration number: CRD42018111308).

### 2.2 Search strategy

Relevant studies in Pubmed, Web of Science, Embase, the Cochrane Library, EBSCO, and CNKI will be searched for studies with language restriction in English and Chinese, which was published from inception to January 1, 2019. Additionally, the reference lists of eligible studies will be screened for other potentially relevant articles. All search results will be imported into endnote software, and duplicate records will be removed by the software. The details of the PubMed full-search strategy are described in Table 1.

**Table 1:**
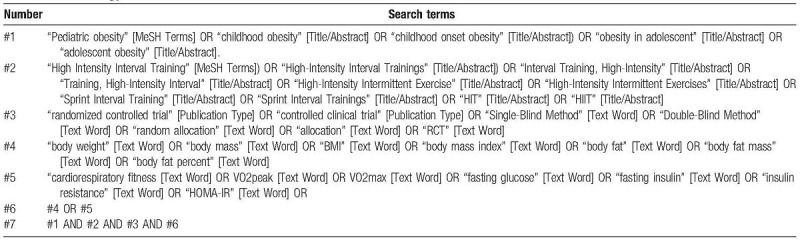
Full-search strategy for PubMed.

### 2.3 Eligibility criteria

#### 2.3.1 Study design

Only randomized controlled trials of HIIT on pediatric obesity will be included, and observational studies, prospective cohort studies, and systematic reviews will be excluded.

#### 2.3.2 Participants

The studies included children who were defined as obese, aged 6 to 18 years, and the definitions of childhood obesity was based on the age and sex-specific body mass index (BMI) cut-off points as indicated by WHO, the Centers for Disease Control and Prevention, or the International Obesity Task Force. The study participants who are normal-weight children, overweight children, and participants aged 6 to 18 years will be excluded.

#### 2.3.3 Interventions

The included studies must involve HIIT and MICT groups, the training frequency of which is more than twice a week for over 4 weeks. HIIT programs involved training at 80% to 100% peak heart rate (HR_peak_) or VO_2peak_^[15]^ for 30 seconds to 4 minutes, interspersed for up to 4 minutes of passive recovery or low-intensity aerobic exercise. MICT programs involved training at 55% to 69% HR_2peak_ or 40% to 59% VO_2peak_ for 20 to 60 minutes.^[16]^ The studies also could be included if HIIT combine with nutritional intervention or lifestyle intervention, but combine with other forms of training, such resistance training, swimming and other ball games will be excluded.

#### 2.3.4 Outcomes

The outcomes will be considered in this review as follows: body anthropometric (eg, body weight, BMI, waist circumference, waist-to-hip ratio); body composition (eg, body fat per cent, body fat mass, visceral fat mass, fat-free mass); glycemic control (eg, blood fasting glucose, blood fasting insulin, glycated hemoglobin A1c [%], Homeostasis model assessment-insulin resistance); blood pressure (eg, systolic blood pressure, diastolic blood pressure); blood lipid (eg, high-density lipoprotein cholesterol, low-density lipoprotein cholesterol, triglyceride, total cholesterol); cardiorespiratory fitness (eg, VO_2peak_).

### 2.4 Selection of studies and data extraction

**Figure 1:**
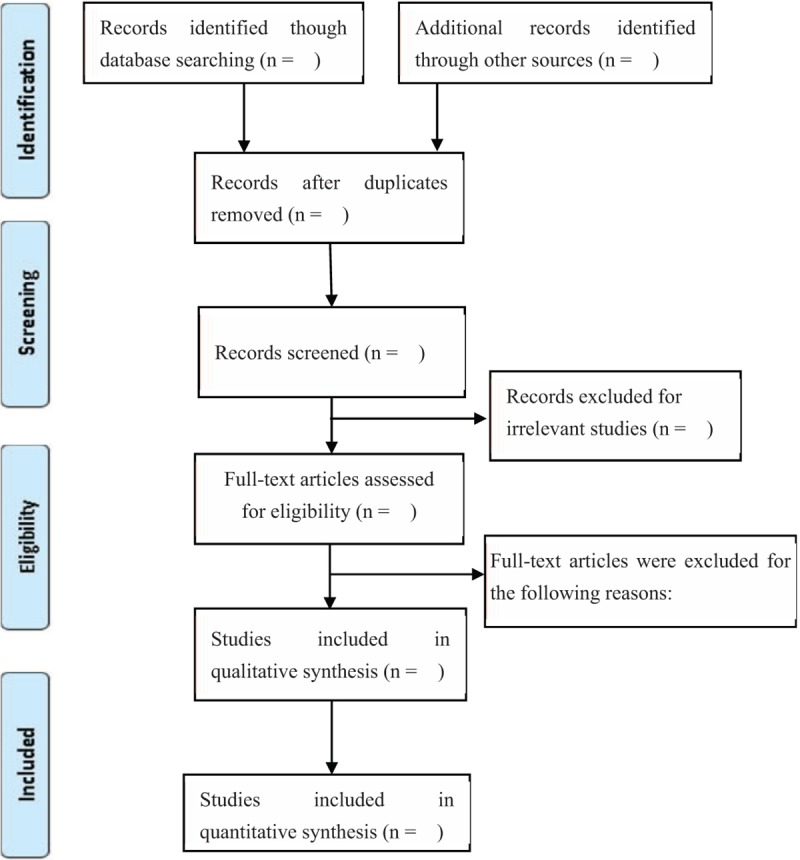
The study selection will be conducted according the PRISMA statement. After removing duplicates form different databases, the titles and abstracts will be evaluated, and then evaluate the full-texts of the remaining the eligibility. Ultimately, qualitative or quantitative evaluation will be conducted.^[17]^ PRISMA = Preferred Reporting Items for Systematic Review and Meta-Analysis.

Two reviewers will independently screen the studies according to the eligibility criteria, and the results are inconsistent when discussed or resolved by a third reviewer. The selection process of eligible papers is shown in Fig. 1.

The data extraction was carried out by 2 independent reviewers in accordance with the standardized sheet recommended by the Cochrane Handbook of Systematic Reviews of Interventions. The following information will be extracted from the included studies: first author's name, year of publication, study design, sample characteristics, training protocol, and main outcomes. If there are any missing data in the studies during the data extraction process, the author will be consulted.

### 2.5 Risk of bias assessment and Grading of Recommendations Assessment, Development, and Evaluation (GRADE) assessment

Two reviewers will independently evaluation of bias of included studies using the Cochrane Collaboration's tools to check for random sequence generation, allocation concealment, blinding, incomplete outcome data, selective reporting, and other bias, each of which makes high-risk, low-risk, and unclear grades.^[18]^

The quality of the evidence will be appraised by 2 independent reviewers, who will use the GRADE system. Disagreements between the 2 reviewers will be resolved by a third reviewer during the evaluation of bias and quality assessment.

### 2.6 Data analysis and synthesis

This study will use the Revman 5.3 software and Stata 12.0 software for data synthesis. The pool effect estimate of the continuous variables will be expressed as mean difference (MD), while the continuous variables in each study are large or are expressed using different units, the standardized mean difference (SMD) will be used. The heterogeneity test between studies will be determined by the *Q* statistic and the *I*^2^ statistic. If *P* > 0.01, *I*^2^ < 50%, it is considered that heterogeneity is low enough to conduct a meta-analysis by a fixed-effect model. If *P* < 0.10, *I*^2^ > 50%, it is considered that there is a large heterogeneity, and a random-effect model will be used. Sensitivity analyses will be further used to reduce heterogeneity by removing studies at high risk of bias or using a 1-by-1 exclusion approach to explore sources of heterogeneity. Sources of heterogeneity will be investigated by meta-regression, which are using age, sex, BMI, exercise intensity, and duration of training as covariates. Funnel plots and Egger test will be used to investigate publication bias.

## 3 Discussion

To the best of our knowledge, this is the first systematic review and meta-analysis protocol to compare HIIT and MICT on fat loss and cardiometabolic health in pediatric obesity. Completing this protocol will determine whether or not HIIT is a superior modality for obese children and adolescents in reducing body fat and improving cardiometabolic health than MICT. Lastly, we feel that this study may lead to several recommendations, for both parents and researchers, as which is the best exercise modality for obese children and adolescents, and what kind of exercise modality need to be designed in weight loss program.

## Author contributions

Jing-Xin Liu contributed to study conception and design, drafting the submitted article, and critically revising the draft for important intellectual content. Lin Zhu revised the draft critically for important intellectual content and gave final approval of the version for publication. Jia-Min Deng contributed to acquisition, analysis, and interpretation the data.

**Conceptualization:** Jing-xin Liu, Lin Zhu.

**Data curation:** Jia-min Deng.

**Formal analysis:** Jia-min Deng.

**Methodology:** Jing-xin Liu, Lin Zhu.

**Project administration:** Lin Zhu.

**Supervision:** Jing-xin Liu.

**Writing – original draft:** Jing-xin Liu.
